# 

**Published:** 1918-06-01

**Authors:** 


Rates of Subscription: Twelve months, 6/6; Six months, 4/-; three months, 2/-, post free to any address in the United Kingdom. Abroad, Twelve months, 8/8; Six months, 5/-; Three months, 2/6, post free. THE HOSPITAL "Index" to Volume LXIII. October 1917 to March 1918, is now ready, price 3½d. per copy, post free. Address The Manager, The Hospital, "The Hospital" Building, 28/29 Southampton Street, Strand, London, W.C. 2.

**URGENT NOTICE.**

To ensure a copy of The Hospital regularly, readers must place a definite order with their Newsagent TO-DAY. After the middle of June, in consequence of the new Paper Restrictions Order, it will not be possible for Newsagents to stock copies for chance customers.

DO NOT DELAY. ORDER

**"THE HOSPITAL" TO-DAY**

and obtain your copy regularly EVERY THURSDAY.

### POSTCARD PROPAGANDA.

Our National Association for the Prevention of Infant Mortality is lending a hand to the Belgian League for the Welfare of Infancy by its issue of a book of twenty-four detachable postcards with English letterpress, showing phases of work in the centres of Belgium. The most interesting are two cards showing the Mothers' Restaurant founded at Brussels in 1915, a fact creditable to the present rulers. Otherwise the pictures are not particularly interesting, but the low price of 1s. may tempt benevolent people to help a cause and effect an economy at the same time. The idea is one which might be taken up by institutions and charities having picturesque aspects. The booklets may be had from 4 Tavistock Square, W.C., or from Bale and Sons, Great Titchfield Street, W. A note appended shows Belgium's need of effort; 21,000 infants under one year were lost in 1912.

### The Nurse's Charter of Liberty.

The article in "The Economics of Nursing" section on page 164 of last week's issue, entitled "The Nurse's Charter of Liberty" has attracted widespread interest and an expression of regret. Owing to great pressure on our space we were compelled to omit two sentences from "Ierne's" article, which we have pleasure in publishing this week, by request. After reproducing the complete text of the Nurse's Charter of Liberty "Ierne" wrote: "The title which Lord Knutsford confers on these conditions of service is a lofty one, and brings to mind such historic scenes as Runnymede, where the feudal barons of England wrung from a tyrant king an Englishman's right to live." Following "Ierne's" request to explain on what human principle its (the Charter of Liberty's) provisions are based, "Ierne" wrote: "I have in mind a very much more venerable charter of liberty, 'Six days shalt thou labour.' The modern democratic ideal runs 'Five and a half days shalt thou labour.'"—Editor, The Hospital.

### Recent Official Publications.

(To be obtained from H.M. Stationery Office, Imperial House, Kingsway, W.C. 2.)

Ministry of Food. Food and How to Save It. Post free 4d.

National Health Insurance. Special Report Series. No. 13. National Research Committee: An Inquiry into the Composition of Dietaries, with Special Reference to the Dietaries of Munition Workers. Post free 11d.

Twenty-third Annual Report of the Local Government Board for Scotland, 1917. Post free 4d.

Statutory Rules and Orders, 1918. No. 421. Public Health, England. Prevention of Epidemic, Endemic, or Infectious Diseases. Public Health (Cerebro-Spinal Fever) Regulations, April 1, 1918. Post free 1½d.

Infant Welfare in Germany during the War. Report Prepared in the Intelligence Department of the Local Government Board, 1918. Post free 7d.

Charity, Scotland. War Charities Regulations, May 1918. Post free 1½d.

Charity Commission. Supplementary List of War Charities. Post free 7d.

---

Mrs. F. S. Williams, of South Kensington, has bequeathed £5,000 each to the Worcester Infirmary and the hospital at Droitwich, in connection with John's Brine Bath Hospital—£1,000 each to the Cancer Hospital, the London Hospital, and the Worcester Ophthalmic Hospital, and a sum equal to the expenses for six months each to the hospice at Hendon and St. John's Convalescent Home, Malvern, for the relief of poor and distressed clergymen.

### The College of Nursing
### (LIMITED BY GUARANTEE).

**Thursday, June 6, 1918, at 3 p.m.**—Annual meeting (for members only) **at the Royal Society of Medicine**, 1 Wimpole Street, W. Tea at 4.30 P.M.

THE CONFERENCE.

**Thursday, June 6, 1918, 7.30 p.m.**—Session I.: "The Higher Education of Nurses and its Relation to Applicants for Training," by Miss Gullan, Sister Tutor, St. Thomas's Hospital. Discussion to follow. Major-General Sir Berkeley Moynihan, F.R.C.S., in the chair. Miss Daunt, Assistant Matron, Middlesex Hospital, in charge of the session.

**Friday, June 7, 191 8, 3 p.m.**—Session III.: "How the Nursing Profession can Assist in the Scheme of Reconstruction," by Hyslop Thomson, M.D., D.P.H., County Medical Officer of Health, Hertfordshire; "The Future Work of the School Nurse," by C. J. Thomas, M.B., Department of Public Health; "The Tuberculosis Nurse," by Jessie M. Campbell, M.B.; "The Nursing Profession in Relation to the Proposed Ministry of Health," by Colonel Thackray Parsons, M.D., R.A.M.C. Miss Haldane, LL.D., C.H., in the chair. The Hon. Gertrude Best, Assistant Matron St. Thomas's Hospital, in charge of the session.

**The Meetings of the Conference are to be held at the Rooms of the Medical Society of London, 11 Chandos Street, Cavendish Square, W. 1, and are open to all those interested in the nursing profession.**

Hospitality is offered for the night of June 6 to those coming from a distance, provided notice is given to Miss Girdlestone, The College of Nursing, 6 Vere Street, London, W. 1, before Tuesday, June 4, 1918.

**Friday, June 7, 1918, 11 a.m.**—A Private Conference for Matrons (past and present) who are members of the College, will be held at the rooms of the Medical Society of London on "Special and Small Hospitals in Relation to General Training Schools."

### Forthcoming Local Meetings.

Plymouth.—Friday, May 31, at 5.30 P.M., at the South Devon and East Cornwall Hospital, to establish a Centre for Plymouth, the Mayor of Plymouth in the chair. Miss Cowlin, Organising Secretary of the College, will give an address.

Truro.—Saturday, June 1.—A meeting organised by Miss Chaff, Matron of the Royal Cornwall Infirmary, Truro, will be held to establish a Centre for Truro. Miss Cowlin, Organising Secretary of the College, will speak.

### Matrons' and Sisters' Appointments.

Edinburgh, Chalmers Hospital.—Miss A. G. Mann has been appointed sister. She was trained at the Bierley Hall Sanatorium, Bradford; the Kendray Hospital, Barnsley; and the Royal Infirmary, Sunderland.

Epileptic Colony, Alderley Edge.—Miss Elizabeth E. Jowett has been appointed night superintendent. She was trained at Nottingham Infirmary, has since done private nursing in Nottingham and Lincoln, and has also been temporary staff nurse at Nottingham Infirmary and night and day sister at the Guest Hospital, Dudley.

Infectious Diseases Hospital, Barry.—Miss May Davies has been appointed matron. She was trained at Hereford County Hospital and Bristol City Hospital, and has since been matron of Llantrisant Isolation Hospital and health visitor to Ogmore and Garw Urban District Council.

Royal Hospital for Sick Children, Glasgow.—Miss E. Mathews has been appointed night sister. She was trained at Stepping Hill Infirmary, Stockport, and has since been staff nurse and ward sister at the York County Hospital.

**Note.—Particulars of Appointments for insertion in this column will be welcomed.**

### Annual Meetings of Four Children's Hospitals.

### HOSPITAL FOR SICK CHILDREN.

### Annual Meetings.

South London Hospital for Women Clapham Common.—Friday, June 7, at 3.15 P.M., at 16 Carlton House Terrace, S.W., the Marchioness of Londonderry, D.B.E., in the chair. Speakers: the Hon. Sir Arthur Stanley, G.B.E., the Marquis of Londonderry, and Mrs. Scharlieb, C.B.E., M.D.

Queen Charlotte's Hospital N.W.—The Ladies' Association annual meeting at the Hospital, Thursday, June 6, 3 P.M. H.R.H. Princess of Arthur of Connaught, patron, will attend.

